# Effects of sildenafil and/or muscle derived stem cells on myocardial infarction

**DOI:** 10.1186/1479-5876-10-159

**Published:** 2012-08-07

**Authors:** Judy SC Wang, Istvan Kovanecz, Dolores Vernet, Gaby Nolazco, George E Kopchok, Sheryl L Chow, Rodney A White, Nestor F Gonzalez-Cadavid

**Affiliations:** 1 Department of Surgery, Los Angeles Biomedical Research Institute (LABioMed) at Harbor-UCLA Medical Center, Torrance, CA, USA; 2 Department of Internal Medicine, Charles Drew University, Los Angeles, CA, USA; 3 Department of Urology, David Geffen School of Medicine at UCLA, Los Angeles, CA, USA; 4 Western University, Pomona, CA, USA

**Keywords:** Stem cells, Myocardial infarction, Heart failure, PDE5 inhibitors, Fibrosis

## Abstract

**Background:**

Previous studies have shown that long-term oral daily PDE 5 inhibitors (PDE5i) counteract fibrosis, cell loss, and the resulting dysfunction in tissues of various rat organs and that implantation of skeletal muscle-derived stem cells (MDSC) exerts some of these effects. PDE5i and stem cells in combination were found to be more effective in non-MI cardiac repair than each treatment separately. We have now investigated whether sildenafil at lower doses and MDSC, alone or in combination are effective to attenuate LV remodeling after MI in rats.

**Methods:**

MI was induced in rats by ligature of the left anterior descending coronary artery. Treatment groups were: “Series A”: 1) untreated; 2) oral sildenafil 3 mg/kg/day from day 1; and “Series B”: intracardiac injection at day 7 of: 3) saline; 4) rat MDSC (10^6^ cells); 5) as #4, with sildenafil as in #2. Before surgery, and at 1 and 4 weeks, the left ventricle ejection fraction **(LVEF)** was measured. LV sections were stained for collagen**,** myofibroblasts, apoptosis, cardiomyocytes, and iNOS, followed by quantitative image analysis**.** Western blots estimated angiogenesis and myofibroblast accumulation, as well as potential sildenafil tachyphylaxis by PDE 5 expression. Zymography estimated MMPs 2 and 9 in serum.

**Results:**

As compared to untreated MI rats, sildenafil improved LVEF, reduced collagen, myofibroblasts, and circulating MMPs, and increased cardiac troponin T. MDSC replicated most of these effects and stimulated cardiac angiogenesis. Concurrent MDSC/sildenafil counteracted cardiomyocyte and endothelial cells loss, but did not improve LVEF or angiogenesis, and upregulated PDE 5.

**Conclusions:**

Long-term oral sildenafil, or MDSC given separately, reduce the MI fibrotic scar and improve left ventricular function in this rat model. The failure of the treatment combination may be due to inducing overexpression of PDE5.

## Background

Cardiac fibrosis is a major factor of tissue remodeling during myocardial infarction (MI) recovery, heart failure, ischemia reperfusion injury, and in most cardiomyopathies [1]. The excessive extracellular matrix, together with the activated fibroblasts and particularly myofibroblasts responsible for its deposition during tissue remodeling, impair the contractile function of the surviving cardiomyocytes. Fibrosis may even affect the normal ECM/fibroblast interaction in force networking around myocytes and putative electrical coupling of both cell types. The etiology, molecular/cellular pathology, progression, and impact on contractile tissue compliance of cardiac tissue fibrosis, resemble the fibrosis occurring in the arterial bed wall [2,3] and in vascular tissues such as the kidney, skeletal muscle, urogenital organs [4-6], and others, except for the cells that are affected and the functional outcomes.

The current conventional therapy of MI, the modulators of the renin-angiotensin-aldosterone system (RAAS), counteracts fibrosis induced by angiotensin II in parallel to other beneficial effects [7]. A novel antifibrotic and cardiomyocyte protective therapy complementing hemodynamic effects is emerging, i.e., the long-term continuous use of phosphodiesterase 5 inhibitors (PDE5i) [8,9], based initially on the cardiac preconditioning exerted by nitric oxide and its main effector cGMP, presumably through inducible nitric oxide synthase (iNOS) [10,11]**.**

A recent study showed that the PDE 5 inhibitor sildenafil given intraperitoneally daily for 4 weeks after permanent left anterior descending (LAD) coronary artery ligation attenuated the increase in left ventricular end-diastolic diameter in the mouse, and improved fractional shortening, overall survival, infarct size, and apoptotic index [12]. The induction of eNOS and iNOS and reduction of apoptosis by this sildenafil treatment mediated by cGMP-dependent protein kinase (PKG) was abrogated in isolated mouse hearts by the inhibition of ERK phosphorylation [13].

Other studies demonstrated that sildenafil blunted interstitial cardiac fibrosis in MI in the rat [14], that the long-term sildenafil or vardenafil regimen exerted similar effects after ischemia reperfusion injury in rabbits [15], and that tadalafil, a long acting PDE 5 inhibitor, improved left ventricular function and survival during doxorubicin-induced cardiotoxicity [16]. However, it is difficult to compare most ischemia/reperfusion studies, performed with single bolus treatment that exerts transient vasodilation, with chronic treatments modifying the underlying cardiac histopathology. For instance, sildenafil in rats reduced infarct size at 24 hrs and cardiomyocyte/endothelial apoptosis while increasing fractional shortening and ejection fraction at 45 days [17]. The same acute treatment in ischemia reperfusion/injury was applied with tadalafil in mice and rats [18,19].

However, despite sildenafil is an approved treatment for pulmonary hypertension in humans, some results with PDE 5 inhibitors in animal models are inconsistent, apparently dependent on the degree of experimental cardiac stress and remodeling [20].

The antifibrotic effects of chronic treatment with PDE5i that may occur on experimental left ventricle remodeling after MI, resembling the process in non cardiac tissues. Long-term, daily treatment with any one of the three PDE 5 inhibitors, as opposed to sporadic administration to induce penile corporal vasorelaxation and thus erection, prevents and even reverses corporal fibrosis in rat models of vasculogenic erectile dysfunction, a sentinel of cardiovascular disease [21-23]. Clinical application of this chronic PDE 5 inhibitor modality is being considered [24]. The antifibrotic action of PDE 5 inhibitors also operates in rat models of bleomycin-induced pulmonary vascular fibrosis [25], diabetic nephropathy [26], and the Peyronie’s fibrotic plaque [27].

PDE5i may be concurrently administered with stem cells to increase the efficacy of adult stem cell therapy for MI [28]. The combination of sildenafil and adipocyte derived stem cells implanted into the left ventricle of rats with dilated cardiomyopathy increased LVEF and angiogenesis while decreasing cardiac oxidative stress, apoptosis and fibrosis, as compared to the stem cells alone [29]. In vitro pre-conditioning of the same stem cells by sildenafil improved their cardiac repair efficacy in mice with MI [30]. It is possible that PDE5i modulate, through cGMP and PKG, stem cell lineage commitment towards cardiomyocytes [31,32].

Mouse and human skeletal muscle derived stem cells (MDSC) induce angiogenesis, reduce scar formation, and improve LVEF, mainly through VEGF expression, in mouse models of MI [33-35]. In rat models of MI, MDSC were therapeutically superior to myoblasts and comparable to bone marrow stem cells, although it is unclear whether MDSC convert into cardiomyocytes [36]. Skeletal myoblasts have a controversial experimental and clinical efficacy, whereas MDSC by being truly pluripotent, and non-myogenically committed cells, are more promising. However, there are no reports on PDE5i modulation of MDSC.

In this study we aimed to investigate whether: a) chronic daily treatment with oral sildenafil at low dose in rats subjected to MI by permanent ligature of the LAD coronary artery improves LVEF, and reduces collagen deposition, myofibroblast accumulation, and loss of cardiomyocytes in the left ventricle; b) intracardiac implantation of MDSC affects similarly cardiac function and remodeling, and sildenafil stimulates these effects.

## Methods

### Ethics

The investigation conforms to the Guide for the Care and Use of Laboratory Animals published by the US National Institutes of Health (NIH Publication No. 85–23, National Academy Press, Washington, DC, USA, revised 1996) and was approved by the IACUC at LABioMed.

### Animal procedures

Male Fisher 344 rats were either 3–4 months old (MDSC isolation), or retired breeders (MI treatments), from Harlan Sprague–Dawley Inc., San Diego, CA, USA under aseptic conditions were anesthetized with isoflurane, intubated, and ventilated to perform a left thoracotomy to expose the heart. MI was induced by permanent ligation of the LAD coronary artery, about 2 mm from the tip of the left auricle, using a 6/0 polypropylene suture (Ethicon, Inc). The chest, muscle, and skin were closed with standard procedures. Rats were allowed to recover from anesthesia, subjected to procedures below and then sacrificed at 4 weeks. Mortality during surgery or for the following 2–3 days was about 30%, and only a couple of deaths occurred thereafter. Replacement rats were added to the study as deaths occurred, so the desired final n = 7-8/group was maintained, unless specified.

**Figure 1:**
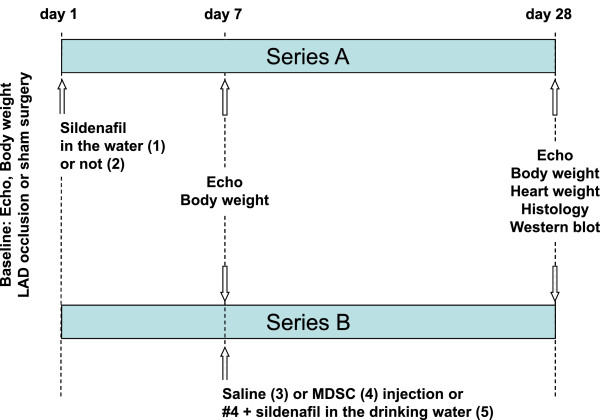
**Experimental protocol.** Myocardial infarction **(MI)** was induced in rats by left anterior descending coronary artery **(LAD)** ligation. Arrows indicate the time points for treatments with sildenafil alone, MDSC, and MDSC + sildenafil, performance of surgical procedure, and measurement of various parameters (listed under each arrow), and final sacrifice.

Rats (final n = 8/group) were randomly divided into five groups: series A: untreated control (group 1); sildenafil (3 mg/kg/day) in the drinking water from day 1 (group 2); series B: aseptic intracardiac injection into the penumbra at day 7, by repeating surgery to expose the heart, of: 0.1 ml saline (group 3); rat MDSC (10^6^ cells in 0.1 ml saline) labeled with the nuclear fluorescent stain 4',6-diamidino-2-phenylindole (DAPI) (group 4); MDSC as group #4, complemented with sildenafil given from day 7 as group #2 (group 5) (Figure 1)

Left ventricular ejection fraction (LVEF) was measured at three stages: a) basal (before surgery); b) 1 week after surgery; and c) before sacrifice, at 4 weeks. Anesthetized animals in the supine or lateral decubitus position were subjected to 2D and M-mode echocardiography (15-MHz linear-array transducer system) under acoustic coupling gel.

### MDSC isolation and culture

MDSC were prepared from the hind limb muscles from the rat [33-36], using the preplating procedure, a validated standardized method for MDSC isolation [37], as in our previous reports [38-40]. Tissues were dissociated using sequentially collagenase XI, dispase II and trypsin, and after filtration through 60 nylon mesh and pelleting, the cells were suspended in Dulbecco’s Modified Eagle’s Medium (DMEM) with 20% fetal bovine serum. Cells were plated onto collagen I-coated flasks for 1 hr (preplate 1 or pP1), and 2 hrs (preplate 2 for pP2), followed by sequential daily transfers of non-adherent cells and re-platings for 2 to 6 days, until preplate 6 (pP6). The latter is the cell population containing MDSC. Cells were then selected using magnetic beads coated with the Sca 1 antibody. Cells were replicated on regular culture flasks (no coating) and used in the 5^th^-10^th^ passage, since the mouse counterparts have been maintained in our laboratory for at least 40 passages with the same, or even increasing, growth rate**.** Flow cytometry was performed to determine whether they were Sca 1+/CD34+/CD44+/Oct 4 cells [40].

### Detection and estimation in tissue sections

At 4 weeks, blood was extracted from anesthetized rats and the animals were sacrificed. The right ventricle and great vessels were trimmed from the heart and the left ventricles were sliced transversally from apex to base into 4 similar height slices numbered from 1 through 4. Slices #2 contained the infarction area and site of MDSC injection and its top ½ region was cryoprotected, embedded in OCT, and used for cryosectioning around the site of cell implantation. The remainder was fixed in 10% formaldehyde fixation for paraffin embedding. In both cases, transverse sections were obtained from apex to base (8 μm). The other left ventricular slices were frozen in liquid nitrogen and stored at −80°C.

The MI area was determined by staining frozen sections with Picro Sirius red, using computerized planimetry for the calculation of the % of infarcted left ventricle [17]. Immunohistochemistry in paraffin-embedded sections was performed [17,28,38-40] for: a) myofibroblasts by α-smooth muscle actin (ASMA) with anti human mouse monoclonal in Sigma kit, 1:2 (Sigma Chemical, St Louis, MO, USA)**;** b) apoptotic index by the TUNEL reaction with the Apoptag kit (Millipore, Billerica, MA, USA); and c) rabbit polyclonal anti-iNOS (Calbiochem/EMD, Brookfield, WI, USA). Cardiomyocyte loss was estimated with a monoclonal antibody against Troponin T-C (Santa Cruz Biotechnology, Santa Cruz, CA, USA)**.** The primary antibodies were detected by the biotinylated anti-mouse IgG (Vector Laboratories, Burlingame, CA, USA), the ABC complex containing avidin-linked horseradish peroxidase (1:100; Vector Laboratories), and 3,3' diaminobenzidine, and counterstaining with hematoxylin. For detecting the implanted DAPI-labeled MDSC, frozen sections were stained for Troponin T, but following with a biotinylated secondary anti-mouse IgG antibody (goat, 1:200, Vector Laboratories) and streptavidin-Texas Red.

The sections were viewed under an Olympus BH2 fluorescent microscope, and quantitative image analysis was performed with ImagePro-Plus 5.1 software (Media Cybernetics, Silver Spring, MD, USA) coupled to a Leica digital bright field/fluorescence microscope/VCC video camera. After images were calibrated for background lighting, integrated optical density (IOD = area x average intensity) was calculated. 6–7 fields were measured per tissue section, with 3–4 sections per specimen, and 8 specimens per group.

### Protein detection and estimations in tissue homogenates

Homogenates from left ventricular region #3 below the infarcted area were obtained in boiling lysis buffer (1% SDS, 1 mm sodium orthovanadate, 10 mm Tris pH 7.4 and protease inhibitors), and centrifuging at 16,000 *g* for 5 min [38-40]. 5–30 μg of protein were run on 4-15% polyacrylamide gels, and submitted to transfer and immunodetection with the antibodies against Troponin T as above, and the additional ones: calponin, mouse monoclonal (Santa Cruz); ASMA, mouse monoclonal (Calbiochem, EMD, San Diego, CA, USA); Von Willebrand factor, rabbit polyclonal (Abcam Inc, Cambridge, MA); PDE5, rabbit polyclonal (Calbiochem); GAPDH, mouse monoclonal (Chemicon, Temecula, CA, USA). Membranes were incubated with secondary polyclonal horse anti-mouse or anti-rabbit IgG linked to horseradish peroxidase (1:2000; BD Transduction Laboratories, Franklin Lakes, NJ, or 1:5000, Amersham GE, Pittsburgh, PA, USA) and bands were visualized with luminol (SuperSignal West Pico, Chemiluminescent, Pierce, Rockford, IL, USA). Quantitative estimation was performed by densitometry, establishing the ratio between the band intensities of each protein against the reference GAPDH value.

### Zymography for MMPs

A serum dilution (5 μg protein) was mixed with equal volumes of zymography sample buffer (125 mM Tris–HCl, pH 6.8, 50% glycerol, 8% SDS, 0.02% bromophenol blue), loaded onto 10% polyacrylamide zymogram gels containing gelatin or casein (BioRad), and electrophoresed with 2.5 mM Tris–HCl, 19.2 mM glycine, 0.01% SDS, pH 8.3, at 100 V [41]. The gels were then equilibrated for 30 min at room temperature with renaturing buffer (2.5% Triton). Zymograms were developed overnight at 37°C in developing buffer, 50 mM Tris–HCl, pH 7.5, 200 mM NaCl, 5 mM CaCl_2_, 0.02% Brij-35. Gels were stained with 0.5% Coomassie Blue for 1 hr, destained with methanol/glacial acetic acid/water (50:10:40), rehydrated in the 5:7:88 mix, and dried. Areas of MMP activity appeared as clear bands. Zymograms intensities were analyzed using NIH Image J.

### Drugs

The following drugs were used: buprenorphine (Reckitt &Colman Products, England) and carprofen (Pfizer, USA) for postoperative pain relief; and sildenafil (Pfizer, USA) dissolved in the drinking water [21-23].

### Statistical analysis

All results are expressed as mean ± standard error of the mean (SEM). The normality distribution of the data was established using the Wilk–Shapiro test. Multiple comparisons were analyzed by a single factor ANOVA, followed by Newman–Keuls multiple comparison test. Differences among groups were considered statistically significant at *P* < 0.05.

## Results

### Effects of chronic sildenafil

**Figure 2:**
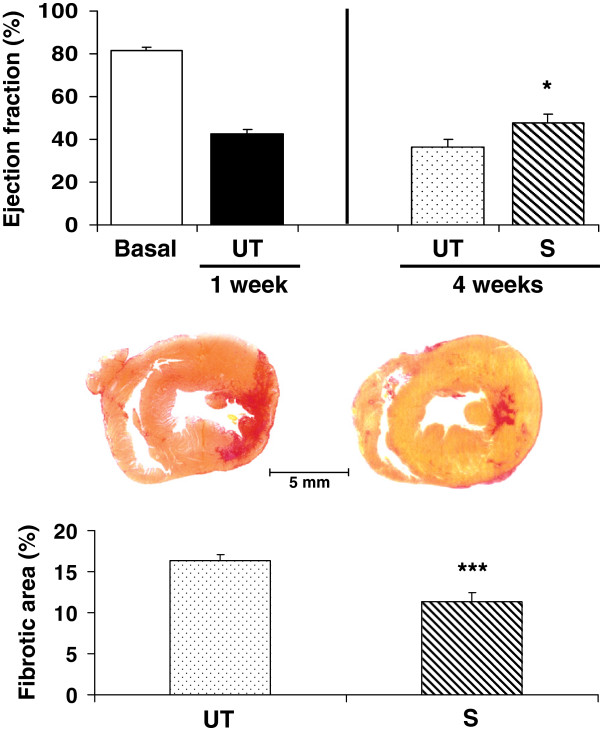
**Long-term oral sildenafil improved left ventricular function and reduced infarction size after LAD occlusion.** Sildenafil was given continuously for 4 weeks in the drinking water (3 mg/kg/day) (n = 8/group). ** *Top:* ** The LVEF was measured before MI (basal), and at 1 and 4 weeks. UT: untreated control; S: sildenafil. ** *Middle:* ** representative micrographs (4 X) for the histochemical detection of collagen by Picrosirius red in paraffin-embedded sections. ** *Bottom:* ** quantitative image analysis of infarction area. Statistical differences are stated for untreated versus basal, and sildenafil versus untreated; *p < 0.05; ***p < 0.005.

We first tested the effects of sildenafil on MI in the rat, at a 3 mg/kg/day given in the drinking water. Figure 2 top shows that this dose moderately (30%) improved the LVEF over the one in the untreated rats (36.6 ± 3.5 vs. 47.8 ± 4.1), and reduced to the same extent the infarction size measured at 4 weeks by quantitative immunohistochemistry for collagen fibers with Picro Sirius red (16.4 ± 0.66 vs. 11.32 ± 1.07), in a region corresponding to the area mainly affected by the LAD occlusion (region #2) (Figure 2 bottom).

**Figure 3:**
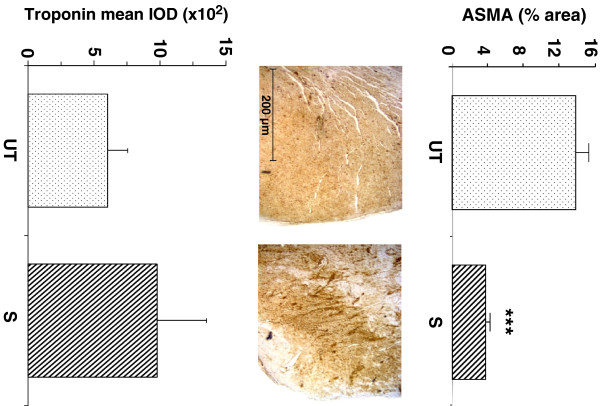
**Long-term oral sildenafil reduced myofibroblast accumulation in the infarction area, but did not significantly counteract the cardiomyocyte loss in this region.** (n = 8/group). Paraffin-embedded sections were used. ** *Top:* ** quantitative image analysis of myofibroblasts by immunohistochemistry for ASMA. ** *Middle:* ** representative micrographs for the immunohistochemical detection of troponin T. ** *Bottom:* ** quantitative image analysis of troponin T. UT: untreated control; S: sildenafil; ***p < 0.005.

The antifibrotic effects of this dose of sildenafil in the rat were confirmed by the considerable 73% reduction of myofibroblasts (13.8 ± 1.5 vs. 3.9 ± 0.4), in the same left ventricular region denoted by ASMA immunostaining (Figure 3 top). iNOS was expressed in this region, as it occurs in most fibrotic processes, but its levels remained unchanged after sildenafil treatment (not shown). There was a non-significant increase by sildenafil on the troponin T content in the same region of the left ventricle (604 ± 152 vs. 979 ± 372), (Figure 3 bottom).

### Effects of MDSC implantation, alone or in combination with sildenafil

**Figure 4:**
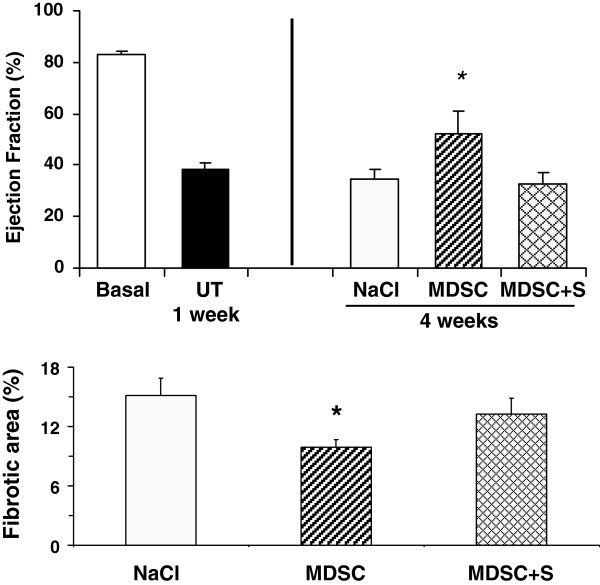
**Intracardiac implantation of MDSC improved the LVEF and reduced infarction size after LAD, but concurrent chronic sildenafil abrogated these effects.** Intracardiac injection of saline or MDSC was conducted at 1 week (n = 7/group). Sildenafil was then given continuously in the drinking water until sacrifice at 4 weeks. ** *Top:* ** Ejection fraction before MI (basal), and at 1 and 4 weeks. NaCl: control injected with saline; MDSC: injection with MDSC; MDSC + S: MDSC with sildenafil. ** *Bottom:* ** quantitative image analysis of infarction area by Picrosirius red histochemistry. Statistical differences are stated for untreated versus basal, and MDSC, and MDSC + sildenafil versus untreated; *p < 0.05.

The intracardiac implantation of homologous (rat) MDSC into the infarcted heart, one week after LAD, improved considerably (50%) LVEF over the value in the saline injected rats (34.6 ± 3.8 vs. 51.9 ± 9.1), (Figure 4 top). However, contrary to expectations, the combination of this treatment with sildenafil at the time of MDSC implantation abrogated the beneficial effects of the cell therapy against the same control (32.6 ± 4.2). This was paralleled by the contrast between a 34% reduction in collagen deposition in the infarcted area in the region around cell implantation exerted by MDSC (15.2 ± 1.7 vs. 9.9 ± 0.7), and the essential disappearance against the same control of this beneficial effect when sildenafil was given concurrently (13.2 ± 1.6), (Figure 4 bottom).

**Figure 5:**
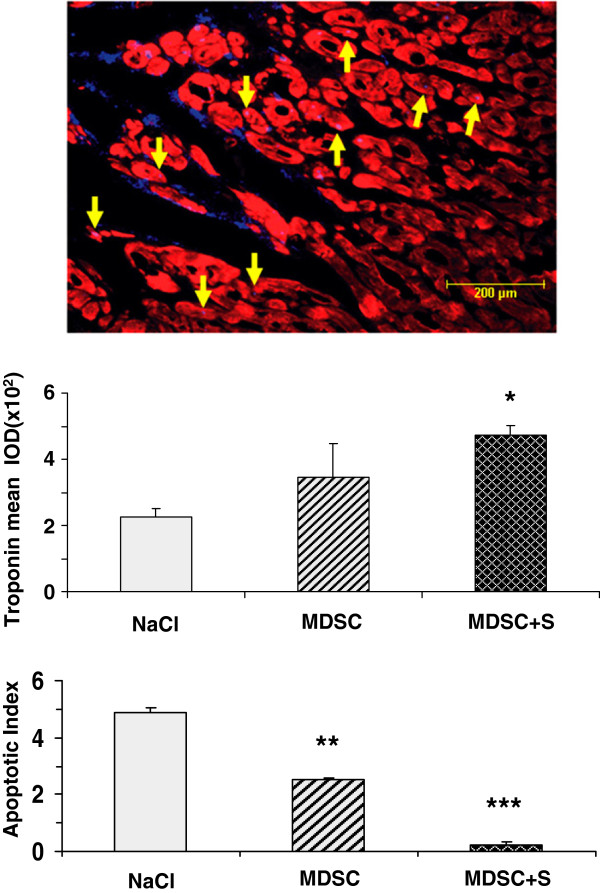
**MDSC implanted in the MI area survived after 4 weeks and reduced the apoptotic index, in a process stimulated by sildenafil that also partially counteracted the cardiomyocyte loss.** (n = 7/group). ** *Top:* ** representative picture of frozen sections from MI regions that received DAPI-labeled MDSC, visualized with blue (DAPI) and red (Texas red) fluorescence filters for implanted nuclei and troponin-T stained cardiomyocytes, respectively. Arrows: cardiomyocyte/MDSC nuclei overlapping. ** *Middle and lower panels:* ** quantitative image analysis by Troponin T and TUNEL (apoptosis) immuno-histochemistry in paraffin-embedded sections, respectively. Abbreviations as in Figure 4. Statistical differences are stated for untreated versus basal, and MDSC, and MDSC + sildenafil versus untreated; *p < 0.05; **p < 0.01;***p < 0.005.

The DAPI-labeled nuclei of the implanted MDSC persisted after 4 weeks in the infarction region and were mostly from cells engrafted in the interstitial connective tissue. A few appeared to overlap the cardiomyocytes identified by troponin T immunofluorescence staining, but this is insufficient to ascertain whether MDSC converted into cardiomyocytes (Figure 5 top). There was a non-significant increase in troponin T in the left ventricle by MDSC, and a higher (30%) and significant increase by the combination of MDSC and sildenafil, as measured by quantitative immunohistochemistry in comparison to the saline injected control (347 ± 99 and 474 ± 27 vs. 227 ± 24) (Figure 5 middle). Matching this cardiomyocyte protection, the apoptotic index was reduced by MDSC by 49% and to virtually negligible levels by the combination with sildenafil (2.5 ± 0.2 and 0.2 ± 0.1 vs. 4.9 ± 0.2) (Figure 5 bottom).

**Figure 6:**
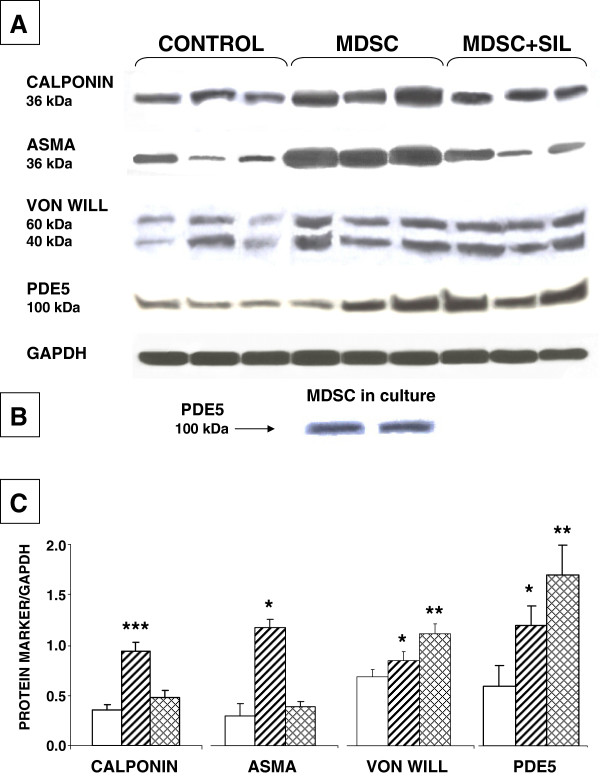
**Implanted MDSC stimulated angiogenesis and reduced myofibroblasts, without affecting PDE 5 expression, while concurrent sildenafil protected the endothelium but increased myofibroblasts and upregulated PDE5.** (n = 7/group). Protein extracts were obtained from region #3 adjacent to the infarction area and subjected to western blot analysis. ** *A:* ** representative immunoblots (n = 8), indicating band sizes. ** *B:* ** PDE 5 assayed in MDSC cultures in duplicate. ** *C:* ** Densitometric values corrected by GAPDH. Statistical differences are stated for untreated versus basal, and MDSC, and MDSC + sildenafil versus untreated; *p < 0.05; **p < 0.01;***p < 0.005.

The modest increase in troponin seen in the infarction area (region #2) by the treatment with MDSC or MDSC + sildenafil was accompanied by an approximately 25% increase in the expression of the 41 kDa troponin band estimated by western blot in the adjacent non infarcted region closer to the base (#3) (1.6 ± 0.2 and 1.5 ± 0.2 vs. 1.3 ± 0.1), but the change was non-significant. In contrast, there was a significant increase by MDSC of the smooth muscle cells (SMC) (1.0 ± 0.1 vs. 0.4 ± 0.1), and endothelial cells (0.9 ± 0.1 vs. 0.7 ± 0.1), represented respectively by calponin and von Willebrand proteins, as an indication of angiogenesis (Figure 6). Although MDSC + sildenafil treatment increased endothelial content, it did not affect significantly calponin. In turn, ASMA expression, a marker of myofibroblasts also shared by SMC was increased by MDSC (1.2 ± 0.2 vs. 0.3 ± 0.1), and sildenafil supplementation only slightly increased ASMA. However, none of the treatments changed significantly the arbitrary, relative ASMA/calponin ratio (1.2 and 0.9 respectively, vs. 0.9 in the control), thus suggesting that the myofibroblast content in region #3 was not changed.

To investigate whether some of the effects exerted by concurrent sildenafil could be due to an increase in PDE 5 protein that would counteract the inhibition of its activity by the drug, PDE 5 was also estimated (Figure 6). That this was the case was shown by the significant increase of PDE 5 levels by MDSC + sildenafil, but not by MDSC alone in comparison to the saline injected rats (1.7 ± 0.3 and 1.2 ± 0.2, respectively, vs. 0.6 ± 0.2). PDE 5 expression was also detected in the MDSC in culture. The magnitude of all the observed changes by western blot assays in the area adjacent to the infarct is likely lower than in region #2 used for histochemical evaluation of the infarct area.

**Figure 7:**
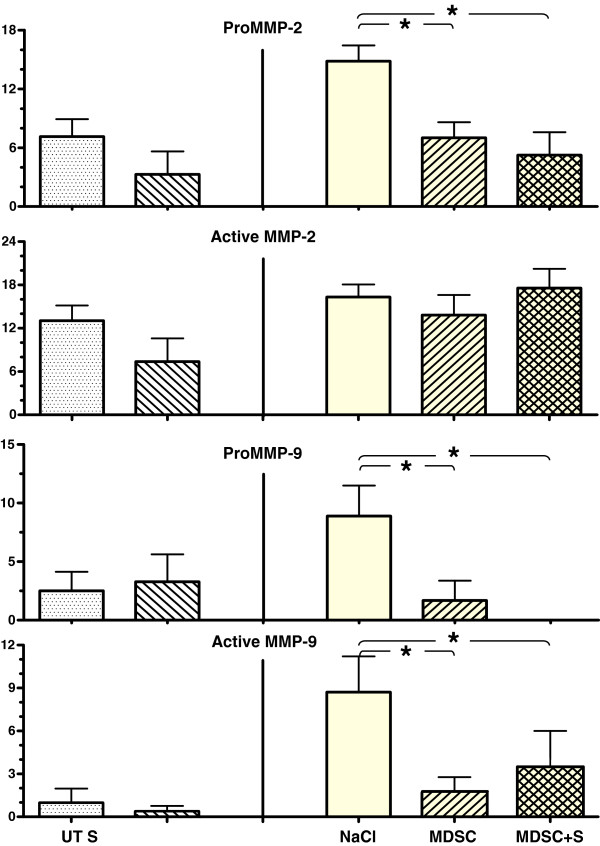
**Implanted MDSC reduced left ventricular remodelling, as indicated by the decrease of both pro MMP-2 and 9 in serum, and concurren**t **long-term oral sildenafil, did not modified these effects.** MMPs were analyzed by zymography (n = 7/group). The Y axes indicate relative densitometric intensities. Abbreviations as in Figures 1 and 3; *p < 0.05.

The relative effects of sildenafil or MDSC alone, or in combination, on left ventricle remodeling were also assessed by determining the release of MMPs to the circulation, using zymography to estimate the levels of the pro-enzymes and processed MMPs. Only the gelatinases MMP-2 and −9 were detected in serum. MDSC reduced significantly the levels of serum pro-MMP-2 (7.0 ± 1.6 vs. 14.8 ± 1.6) and −9 (1.7 ± 1.7 vs. 8.9 ± 2.6) and of active MMP-9 (1.7 ± 1.1 vs. 8.7 ± 2.5) in comparison to the saline injection, but the concurrent administration of sildenafil did not alter these effects (Figure 7).

## Discussion

This study aimed to address the issue on whether concurrent long-term daily administration of low doses of oral PDE 5 inhibitors, compatible with standard on demand clinical use, can stimulate the potential antifibrotic and antiapoptotic effects of stem cells, in this case MDSC, on MI repair, thus extending to this condition prior similar studies with PDE 5 inhibitors alone on the vascular bed and in urological organs, and even in avascular tissues [6,16,21-23,27]. First, we have shown that oral sildenafil given alone (no MDSC) to the rat for 4 weeks post-MI, acts as expectedly, by moderately increasing the LVEF and troponin recovery in tissue sections in the left ventricular region around the infarction, and reducing the fibrotic area and myofibroblast infiltration. Second, MDSC given alone (no sildenafil) acted similarly, while also reducing apoptosis measured by TUNEL, enhancing angiogenesis (SMC content) assayed by western blot, and lowering tissue remodeling as indicated by pro-MMP 2 and 9 and active MMP 9 levels in serum. Third, the concurrent long-term administration of MDSC + sildenafil to rats with MI intensified as expected the antiapoptotic, and cardiomyocyte and endothelial protective effects of the separate MDSC and sildenafil treatments, and preserved the serum MMP pattern of the rats receiving MDSC.

However, unexpectedly the MDSC + sildenafil combination treatment inhibited the improvement of the LVEF and the reduction of the fibrotic area by MDSC or sildenafil alone, and the increased angiogenesis (measured by SMC content) by MDSC. We postulate that this abrogation by the combination treatment of some of the beneficial effects on cardiac tissue and function exerted by each independent treatment is due in part to the observed upregulation of PDE 5 expression (that would counteract the inhibition of PDE 5 enzyme activity by sildenafil alone).

The dose of oral sildenafil for the current rat study, 3 mg/kg/day, was selected to be clinically translatable to humans, and also to be compatible with the goal of modulate the differentiation of stem cells given concurrently [42]. It is double the daily dose given for 4 weeks either IP for MI in the mouse [12], or orally, concurrent with MDSC, as antifibrotic in the rat corpora cavernosa [43]. This dose comparison is likely to be reflected in the respective sildenafil blood concentrations, since neither the oral vs. IV or IP administration [44] nor the small difference in rat/mouse surface/weight ratios [45] are expected to affect considerably the proportional pharmacokinetics. Translated to the human based on the rat/human surface correction factor [21,23,45], our oral dose would be roughly equivalent to about 30–40 mg/day, or slightly less than the usual oral dose given sporadically on demand to induce penile erection through corpora cavernosal vasodilation. Other studies on MI in the rat [14] have used much higher doses of sildenafil (100 mg/kg/day), but when they are translated to human treatment they exceed considerably (more than tenfold) the clinical doses.

The beneficial effects of PDE5is for experimental ischemia/reperfusion, cardiac hypertrophy, and heart failure [9-19,46] have been ascribed to mechanisms as varied as nitric oxide generation by upregulation of iNOS or eNOS, protein kinase C activation, opening of mitochondrial ATP-sensitive potassium channels, or inhibition of the RhoA-Rho kinase pathway in cardiac tissue, or even distal effects reducing peripheral resistance and aortic and large artery stiffness. However, we believe that the improvement of the LVEF by our selected chronic daily dose of sildenafil resulted from the expected antifibrotic action of PDE5is that replicated the inhibition of collagen deposition, myofibroblast accumulation, and the preservation of key functional cells under various tissue damage conditions previously described in non cardiac tissues [4,6,21-23,27]. This is essentially due to the inhibition of collagen synthesis, and myofibroblast differentiation, and in certain cases, of apoptosis that by counteracting fibrosis not only helps to protect the normal cardiac tissue composition, but also the normal ECM/fibroblast interaction in force networking around the myocytes and putative electrical coupling of both cell types [1].

Our results on the reduction of MI scar size coincide with the two long-term experimental studies of cardioprotection in mice and rats after permanent LAD occlusion by PDE5i given IP [12,17]. The caveat is that in the latter studies measurements were done at 24 hrs when the effects are due to rapid vasodilation and related mechanisms instead of the long-term antifibrotic action. Myofibroblast accumulation, troponin T loss, angiogenic markers, or MMPs in serum were not reported in those papers.

The lack of intensification of iNOS expression in the rat post-MI cardiac tissue by sildenafil does not agree with what was observed in the mouse in this condition and in ischemia reperfusion injury [12,13], and even in other organs [21-23], suggesting that studies on the time course of iNOS blockade or overexpression on the cardioprotective effects of chronic sildenafil in MI are needed to clarify these discrepancies. This is of interest, considering that although in other tissues fibrosis was exacerbated by blocking iNOS by long-term administration of the iNOS inhibitor L-NIL or by its genetic inactivation in the iNOS ko mouse [4,46,47], in MI the role of iNOS in fibrosis, vis-à-vis eNOS, is confusing, as evidenced by various reports claiming deleterious, protective, or no effects [47]. This may result from the opposite actions of iNOS in the early inflammatory remodeling phase as compared to the subsequent fibrogenesis.

The improvement of cardiac function, reduction of fibrotic scar, and cardiomyocyte preservation by MDSC implantation into the MI area are in agreement with previous results in mice [33-35] and in rats [36], although it is not clear whether there is some conversion of the engrafted cells into cardiomyocytes that does occur with myoblasts or satellite cells, or this is exclusively due to trophic effects such as the stimulation of angiogenesis. The latter seems to have occurred in the present work, as judged by the observed increase in SMC and endothelial markers.

The common denominator of these effects of sildenafil is the expression of PDE5 in both the human left and right ventricle, specifically in smooth muscle and endothelial cells, and in the cardiomyocytes themselves, which is considerably increased in end-stage ischemic cardiomyopathy, and the assumption that this may contribute to at least right ventricular heart failure [20]. The observed upregulation of PDE5 in the MDSC/long-term sildenafil combination may explain the loss of efficacy of MDSC in improving LVEF and reducing scar size, since this may cause tachyphylaxis [20,48]. This PDE5 upregulation has been postulated to occur in the penile smooth muscle due to the presence of cGMP-responsive elements in the PDE5 gene promoter [49], but has not been observed in vivo [50]. Our assumption would require on one side that sildenafil modulates MDSC lineage commitment towards a fibrotic phenotype and myofibroblast formation, and on the other side that the PDE5 upregulation itself occurs in the MDSC or their differentiation, since in the absence of MDSC sildenafil was moderately effective. The MDSC myofibroblast differentiation was suggested previously [51], assuming that the release of local environmental stimuli after muscle injury triggers the differentiation of MDSC into fibrotic cells, thus illustrating the importance of controlling the local environment within the injured tissue to optimize regeneration via the transplantation of stem cells. We have shown this differentiation in vitro [40]. This process also occurs with mesenchymal cells, even in the absence of injury [52]. Moreover, endogenous cardiac stem cells originate fibroblasts, which are essential for proper tissue repair, but also myofibroblasts whose accumulation may lead to inadequate scar formation [53]. In turn, sustained high cGMP levels induced by PDE5is, in the absence of PDE5 upregulation, reduced myoblast formation [54], so that the reverse, i.e. the decrease of cGMP by high PDE5, may trigger this differentiation. It is of interest that sildenafil was effective in potentiating the efficacy of adipose-derived mesenchymal stem cells on cardiac repair in rat dilated cardiomyopathy [29], but it reduced the efficacy of MDSC in tissue repair, in this case in corporal penile fibrosis and loss of SMC subsequent to nerve damage [43], suggesting that various types of stem cells may react differently to PDE5i in terms of their repair capacity.

Altogether our results confirm in the rat that daily oral sildenafil at low dose and MDSC exert separately a modest cardioprotection post-MI by ameliorating the infarction scar formation and remodeling, but the in vivo combination of these treatments, at least in the rat, is counteractive. Further research is needed to identify sildenafil regimens that may not induce the PDE5 upregulation, or alternatively by overriding the higher PDE5 levels by an efficient inhibition of enzyme activity. In vitro preconditioning of stem cells with PDE5i prior to implantation has just been shown, in this case with sildenafil and adipose-derived stem cells, to reduce in the mouse post-MI cardiomyocyte apoptosis and fibrosis, possibly by improving stem cell survival and paracrine effects by secretion of growth factors [30] . This strategy would avoid the in vivo sildenafil/stem cell interaction in the host cardiac tissue setting, while preserving the beneficial effects on stem cell trophic effects and/or lineage commitment, even in the absence of a direct protective action by sildenafil on cardiac tissue.

The design of the current work was restricted to the five arms already described, in order to simplify it. However, once the sildenafil dosages and times of administration are optimized, the selected treatment should be compared with the conventional RAAS therapy [7,55,56]. This may involve an angiotensin II type I receptor blocker or a type 2 receptor stimulator, or an angiotensin-converting enzyme inhibitor, based on their well known antifibrotic, anti-inflammatory, and cardiomyocyte protection effects, and also in combination with MDSC. In fact, it is known that RAAS modulators can inhibit or stimulate cardiovascular progenitor functions, even if the overall picture is not yet clear [57]. For instance, although AT(1) receptor blockade and ACE inhibition stimulate proliferation and differentiation of endothelial progenitor cells (EPC) and angiogenesis, Ang-(1–7) that behaves similarly towards EPC may either inhibit or stimulate angiogenesis according to dosages**.** There are no reports on the modulation of implanted stem cells by these agents. Similarly, no studies with a RAAS/PDE5i combination have been reported, even if combo approaches in the absence of stem cells with AT(1) blockers and ACE inhibitors are being tested [58]. Therefore, the optimal MDSC/sildenafil combination should also be tested against an MDSC/RAAS combination to assess which antifibrotic/pro-differentiation approach may be more efficacious.

## Competing interests

The authors declare that they have no competing interests.

## Authors' contributions

Experiment design and manuscript drafting: NFGC with the assistance of RAW. Animal experiments: JSCW, IK, SLC. Ejection fraction measurements: JSCW, GEK, IK, SLC. Laboratory assays: JSCW, IK, DV, GN, SLC. All authors read and approved the final manuscript.
